# Pervasive Games for Sexual Health Promotion: Scoping Literature Review

**DOI:** 10.2196/58912

**Published:** 2025-01-15

**Authors:** Claudio Rubio, Felipe Besoain

**Affiliations:** 1 Faculty of Engineering Universidad de Talca Curicó Chile; 2 Department of Interactive Visualization and Virtual Reality Faculty of Engineering University of Talca Talca Chile

**Keywords:** serious games, promotion, ubiquitous technologies, healthy behaviors, HIV, sexually transmitted infection, STI, scoping review, mobile phone

## Abstract

**Background:**

Serious games play a fundamental role in promoting safe sexual behaviors. This medium has great potential for promoting healthy behaviors that prevent potential risk factors, such as sexually transmitted infections, and promote adherence to sexual health treatments, such as antiretroviral therapy. The ubiquity of mobile devices enhances access to such tools, increasing the effectiveness of video games as agents of change.

**Objective:**

In this scoping review, we aimed to (1) identify the extent to which pervasive games have been used in the field of sexual health, (2) determine the theories used in the design and evaluation of pervasive games for sexual health, (3) identify the methods used to evaluate pervasive games for sexual health, and (4) explore the reported benefits of using pervasive games for sexual health.

**Methods:**

Following the PRISMA-ScR (Preferred Reporting Items for Systematic Reviews and Meta-Analyses extension for Scoping Reviews) methodology, we conducted a comprehensive literature search in the Web of Science, Scopus, IEEE Xplore, and ACM databases for articles published between January 1, 2000, and August 4, 2024. Included articles were published in English between 2000 and 2024 and involved the design, implementation, or evaluation of a ubiquitous video game focused on promoting safe sexual behaviors, with qualitative and/or quantitative results based on theory-based techniques and ubiquitous technologies. Review articles, conference papers, or books without available data or quantitative or qualitative results were excluded.

**Results:**

We screened 521 of 612 articles (85.1%) after removing duplicates. After the title and abstract review, 51 (9.8%) articles were assessed for eligibility, and 30 (5.8%) articles meeting the criteria were studied and evaluated in depth. The results suggested that the use of pervasive video games has a positive impact on promoting safe sexual behaviors. This is enhanced by the effectiveness of theory-based techniques and the use of mobile technologies as developmental factors that drive the gaming experience. The results indicated that this domain is a growing field that should not be ignored.

**Conclusions:**

The literature showed that pervasive video games have been effective in promoting safe sexual behaviors. Substantial growth has been seen in scientific community interest in researching this domain; nevertheless, there is still much to work on. In this context, we advocate for the standardization of design, implementation, and experimentation as essential phases in creating video game experiences. These 3 fundamental aspects are critical in the development of video game–based studies to ensure the reproducibility of experiments.

## Introduction

### Background

The global effort to combat sexually transmitted infections (STIs), including HIV and viral hepatitis, faces significant hurdles in promoting sexual health safety. These infections account for 2.3 million deaths and 1.2 million cancer cases annually. Daily, there are >1 million new STI cases, with 4.5 million individuals contracting HIV and hepatitis B and C annually [1,2]. Despite progress in HIV treatment, 2021 witnessed 1.5 million new HIV infections and 650,000 AIDS-related deaths, underscoring the persistent challenge HIV presents [3]. By the end of 2022, the population that tested positive for HIV reached approximately 39 million, with a notable 38% reduction in new infections since 2010; yet, HIV infection remains a major health issue [4]. Statistics also reveal alarming rates of STI transmission, with >1 million new infections daily, 374 million new cases of curable STIs per year, >500 million people living with herpes simplex virus, and human papillomavirus (HPV) causing >311,000 cervical cancer deaths annually [5]. These figures highlight the critical need for intensified global action aimed at achieving the diagnosis, treatment, and suppression goals for STIs by 2030 [6].

One current focus of these strategies is to promote sexual education and sexual well-being through access to reliable and detailed information covering topics related to sexuality and the risk of conditions such as HIV, STIs, and HPV [2]. However, despite significant progress in raising awareness and promoting safe sexual practices, effectively implementing these strategies presents notable challenges. Cultural taboos and sensitivities associated with traditions can complicate conversations about sexuality in some communities [7]. In addition, conservative roles and gender expectations among teenagers create barriers that limit access to sexual education, especially in areas where health professionals and parents are the primary providers of information [8]. As a result, there is a lack of comprehensive sexual education that provides teenagers with the necessary knowledge for a safe and healthy sexual life [9].

In a world where rapid and efficient access to information has become indispensable, ubiquitous and pervasive technologies have become a necessity. Ubiquitous computing focuses on the seamless and natural integration of technology into the environment, aiming to be omnipresent but noninvasive [10]. In contrast, pervasive computing focuses on the immediate use of processed information through the use of mobile devices and wireless communication [11,12]. Both concepts share the goal of making technology accessible and useful in everyday life but differ in their approach and application [13].

With the proliferation of mobile devices, the use of pervasive apps has become a standard, primarily due to the portability they offer to the users, enabling efficient access to knowledge through any device, at any time, and anywhere [14]. This advancement has created a unique space for innovative software development in the health sector [15]. Apps in this area have been vital for health promotion, and due to their omnipresent nature, they have integrated into people’s daily lives [16].

For example, studies have been conducted on the use of mobile devices for the continuous monitoring, diagnosis, and treatment of mental health conditions [17,18] as well as to improve adherence to antiretroviral therapy (ART) in young people with HIV [19]. A review of serious video games for the prevention of HIV concluded that these games can be innovative and effective, as they provide interactive experiences with realistic scenarios, establish an agency in a safe virtual environment, and allow individuals to practice and develop skills without social stigma [20]. Another review highlighted the effectiveness of serious games on mobile platforms and the application of gamification techniques as innovative strategies in the prevention and care of HIV or AIDS, emphasizing their cost efficiency compared to long-term treatment options [21]. A third review in the field emphasized the use of persuasive games as tools for promoting healthy behaviors in domains such as physical activity, disease control, nutrition, and environmental sustainability, among others. Besides highlighting the importance of basing these games on behavioral theory, it suggested the need for longitudinal studies and more detailed evaluations of persuasive strategies to optimize the design of future persuasive games [22].

These studies underscored the value of serious games as effective tools for promoting adherence to healthy behaviors. Through simulations, these games provided exceptional opportunities for learning and skill development, becoming optimal means for awareness, education, and treatment of diseases. They also offered a pleasant and motivating way to interact with complex topics [23], including learning [24], behavior modification [25], and the promotion of healthy habits [26].

However, in health promotion through pervasive apps, it is crucial to distinguish between serious games and gamified apps. Serious games naturally present themselves as real games, are focused on improving performance or learning, and are designed to drive behavioral and attitudinal changes, offering an immersive experience where the act of playing itself is central [22,27]. In contrast, gamified apps incorporate ludic elements into traditionally nongaming environments to create an experience similar to a video game [28], with the aim of motivating and engaging users in the process of attitudinal change [29]. While they apply ludic aspects, they are not games themselves but tools that enhance the user experience by integrating game elements to enrich conventional activities [21,28]. Although both methods can be effective in shaping attitudes, this review focuses on serious games due to the significant difference in the gaming experience they offer compared to gamified systems.

### This Study

This scoping literature review focused on exploring the intersection between theory-based techniques (persuasive, cognitive, or behavioral) applied in serious games and the use of pervasive media as integral components of the gaming experience. Exploring this relationship is crucial to identify the strengths, weaknesses, gaps, and opportunities that these media offer in promoting safe sexual behaviors. This study aimed to provide a deeper and more detailed perspective on how pervasive mobile games can effectively contribute to sexual health promotion, attitude change, and sexual education in our society. Building on gaps observed in previous works that studied the use of video games for promoting safe sexual behaviors [20-22], this study sought to answer 4 research questions (RQs): RQ1—How have pervasive games been used in the field of sexual health? RQ2—What methods and technologies have been used to design pervasive video games for sexual health? RQ3—What theories are used in the design and evaluation of pervasive games for sexual health? and RQ4—What methods, along with their benefits, have been used to evaluate pervasive games for sexual health?

## Methods

### Overview

This scoping review followed the established methodological framework proposed by Arksey and O’Malley [30]. We adopted the PRISMA-ScR (Preferred Reporting Items for Systematic Reviews and Meta-Analyses extension for Scoping Reviews) guidelines [31]. The completed checklist is provided in Multimedia Appendix 1. We did not preregister for this scoping review because of its descriptive and exploratory nature.

### Search Strategy

We explored 4 databases: Web of Science, Scopus, IEEE Xplore, and ACM. Web of Science and Scopus were chosen due to their ability to provide a comprehensive view of the evolution of serious games focused on promoting healthy behaviors and their relationship with pervasive games. In addition, IEEE Xplore and ACM were included due to their specialized focus on engineering and technology. The time frame analyzed spans from January 1, 2000, to August 4, 2024. In addition, we used the population, intervention, comparison, and outcomes (PICO) structure to define a suitable search string [32]. Textbox 1 presents the keywords of the PICO structure.

**population, intervention, comparison, and outcomes analysis with search terms.**

**Population**

- Video games focused on promoting safe sexual health
- Terms: ((videogame OR games) AND (Health* OR well* OR self* OR care*) AND ((study OR intervention) AND (sex* OR STI)))

**Intervention**

- Design, implementation, or evaluation of pervasive video games and use of theory-based techniques
- Terms: ((videogame OR games) AND (persuasive OR persuasion OR theories OR strategies OR behav* OR change OR changing OR promote OR promotion OR promoting OR attitude OR prevent OR prevention OR preventing OR improve OR improving))

**Comparison**

- No specific direct comparison specified
- Terms: not applicable

**Outcomes**

- Qualitative and quantitative analysis of obtained data
- Terms: ((videogame OR games) AND ((study OR intervention) AND (sex* OR STI)))

We connected these concepts using the “AND” and “OR” operators, resulting in the following search string: ((videogame OR games) AND (persuasive OR persuasion OR theories OR strategies OR behav* OR change OR changing OR promote OR promotion OR promoting OR attitude OR prevent OR prevention OR preventing OR improve OR improving) AND (pervasive OR ubiquitous OR mobile OR smartphone OR omnipresent OR interactive OR presence) AND (design OR implement OR evaluation OR development OR technology OR technologies) AND (Health* OR well* OR self* OR care*)) AND ((study OR intervention) AND (sex* OR STI)). The complete search strategy, including a full list of search terms, is provided in Multimedia Appendix 2.

### Eligibility Criteria

To systematically discern between eligible and ineligible articles for review, we defined a set of inclusion and exclusion criteria. Textbox 2 summarizes the criteria used.

**Inclusion and exclusion criteria.**

**Inclusion criteria**

- Language: articles must be written in English
- Date: articles published between January 1, 2000, and August 4, 2024
- Database: articles must belong to the databases Web of Science, Scopus, IEEE Xplore, or ACM
- Document type: article or protocol
- Study type: studies based on the design, implementation, or evaluation of pervasive video games
- Health area: studies focusing on promoting safe sexual health
- Result type: articles with qualitative and quantitative data analysis.
- Device: specifically, the studies involving mobile devices in the context of promoting safe sexual health through pervasive video games

**Exclusion Criteria**

- Intervention: articles presenting gamified apps not specifically related to the use of pervasive video games for promoting safe sexual behaviors
- Data: studies with unavailable data
- Document type: nonempirical studies such as reviews, conference papers, or books that do not present empirical data
- Result type: articles that lack qualitative or quantitative data analysis
- Empirical: articles that do not focus on the use of video games for promoting safe sexual behaviors and present content relating to topics other than sexual health promotion (eg, nutrition, physical activity, smoking cessation, and mental health)

### Study Selection

We used the Hubmeta platform to facilitate the process of duplicate removal, title screening, and full-text screening [33]. Once the queries were made in the selected databases, the results were imported into the platform and the reviewers did the screening process independently. At the end of the process (title screening and full-text screening), if reviewers disagreed about inclusion, a meeting was held to discuss and review the points from the PICO structure. All disagreements were resolved without the need for a third reviewer. Finally, the included articles were studied thoroughly in search of elements that answer the RQs.

### Data Charting

The final list of data charting elements is presented in Textbox 3, in which we outline the proposed organization for storing the key data from each article reviewed. Data charting for all elements was conducted using a Microsoft Excel sheet. All the categories for different data charting options were initially created based on brainstorming relevant information and then refined with the information of a small subset of articles. Next, data were registered and discussed among the authors involved in the charting process.

**Data charting categorization.**

**Article data**

- Name
- Year of publication
- Database
- Journal
- Type (protocol or article)

**Game characteristics**

- Name
- Topics
- Backstory or objective
- Genres
- Tasks

**Development design**

- Design methodology
- Target audience
- Device
- Inputs
- Behavioral change theories

**Intervention characteristics**

- Research design
- Sample population
- Country
- Mean age and SD
- Duration of dose
- Follow-up

**Experimental data**

- Results (preliminary or significative)
- Evaluation (qualitative, quantitative, or mixed)
- Outcomes (positive, negative, or neutral)

The information is categorized into five main groups: (1) article data, which include general information about each article; (2) game characteristics, which detail the key aspects of the video game; (3) development design, which focuses on the critical aspects of the video game’s development phase; (4) intervention characteristics, which capture data related to the experimental stage of the various studies; and (5) experimental data, which present the results obtained during the experimental process.

Article data include the name of the article, the year of publication, the database from which it was retrieved, the journal, and the type of document (article or protocol). Although protocols only present the experimental design of future research, they were included because they helped identify important studies that were not captured in the initial queries.

Game characteristics capture the name of the video game, the topics addressed, the backstory or objective the character must achieve, the game’s genres, and the tasks the user must complete to finish the experience. It is important to note that a video game can cover multiple topics and belong to more than one genre, so both categories allow for multiple selections.

In development design, the design methodologies guiding the experience were documented, along with the target audience, following the categorization guidelines from the World Health Organization [34,35] and the Centers for Disease Control and Prevention [36]. In addition, the devices for which the experience was developed, the inputs used, and the behavioral change theories that guided the attitudinal change process within the game were recorded. It is worth noting that a video game can incorporate multiple behavioral change theories, so an article might present >1 theory.

Intervention characteristics such as the research design guiding the experience were identified, along with the sample population and its characteristics. The locations of the participants were recorded based on the countries from which they were recruited (if applicable). If an article did not describe participant recruitment, the authors’ countries were recorded based on their affiliations. The mean (SD) ages of participants were obtained from the articles, and when unavailable, they were estimated following the recommendations by Hozo et al [37]. Alternatively, a weighted mean and weighted SD calculation was performed. If it was not possible to obtain the value by any of these methods, the box was marked as not available. The duration of the intervention, including the number of sessions and their length, and the follow-up period were also recorded.

Experimental data store the experimental results obtained from the articles. Since protocols are experimental designs for future studies, they do not provide experimental results and thus do not include this type of information. Articles were first categorized into 2 types: preliminary (those presenting proof of concept and their results) and significant (those conducting statistically significant studies), following the recommendations by Jitmun et al [21]. In terms of evaluation, articles were divided into three categories: qualitative, quantitative, and mixed methods. Finally, outcomes were categorized based on the contributions indicated by the authors as positive, negative, or neutral.

## Results

### Search Results

Our search returned 612 articles (Web of Science: n=150, 24.5%; Scopus: n=384, 62.7%; ACM: n=76, 12.4%; and IEEE Xplore: n=2, 0.3%). Of 612 articles, we found and discarded 91 (14.9%) duplicate articles. Results of the 521 articles reviewed after remove duplicates can be founded in Multimedia Appendix 3. In the title screening, we evaluated the title and abstract of each article. From this step, we discarded 470 (90.2%) of the 521 documents for not meeting the inclusion and exclusion criteria.

The remaining 51 articles were included for full-text screening. After checking the full text of these 51 articles, 24 (47%) were excluded for presenting gamified apps or the central theme of the article not being related to the use of serious games for promoting safe sexual behaviors.

**Figure 1:**
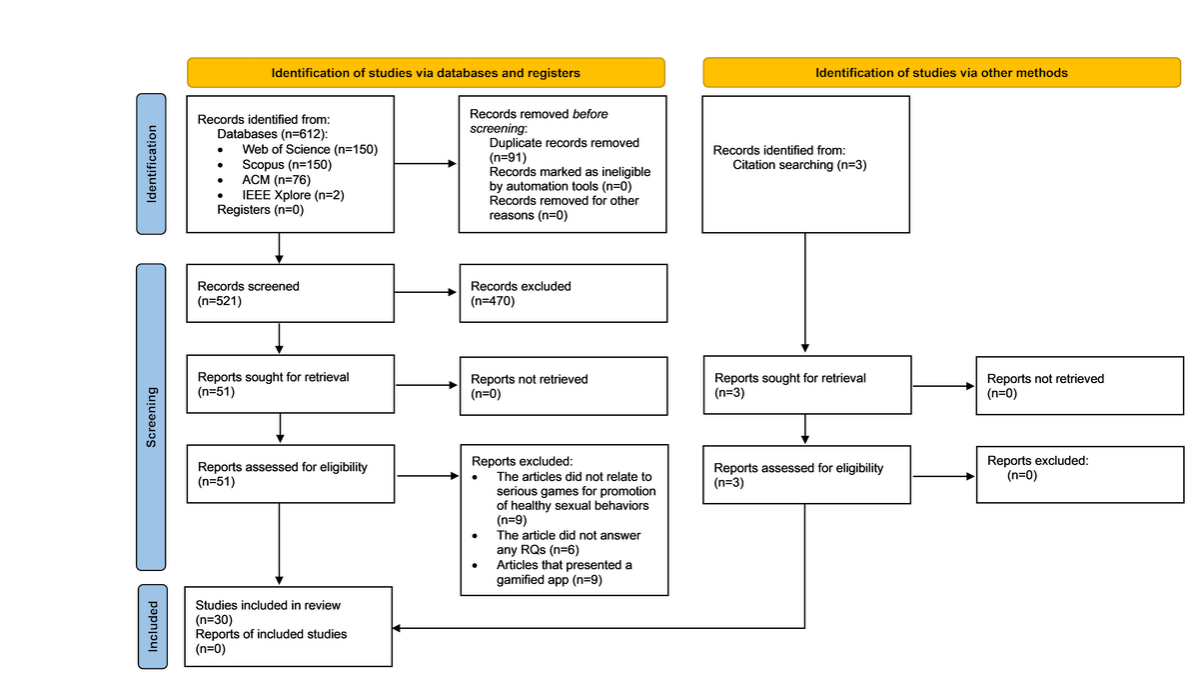
PRISMA-ScR (Preferred Reporting Items for Systematic Reviews and Meta-Analyses extension for Scoping Reviews) extension for scoping reviews flow diagram. RQ: research question.

Finally, we identified 27 articles after this process, including 22 (82%) research articles and 5 (18%) protocols. Following the 5 protocols retrieved, we identified 3 additional research articles related to the protocols that were not retrieved by the original queries. Accordingly, the final number of included studies was 30. Figure 1 [38] presents the PRISMA-ScR flow diagram. The details of the papers’ findings are provided in Multimedia Appendix 4.

### General Screening

We observed that the most common journal where articles were published was *JMIR Serious Games* (8/30, 27%), followed by journals *JMIR Research Protocols* (3/30, 10%) and *Games for Health Journal* (3/30, 10%). The third position was shared by journals *JMIR mHealth and uHealth* (2/30, 7%), *BMC Public Health* (2/30, 7%), and *JMIR Formative Research* (2/30, 7%). The remaining articles (10/30, 33%) were published in multiple journals, each with only 1 article. However, we did not find any studies published from 2000 to 2009, 2011 to 2014, or in 2017.

**Figure 2:**
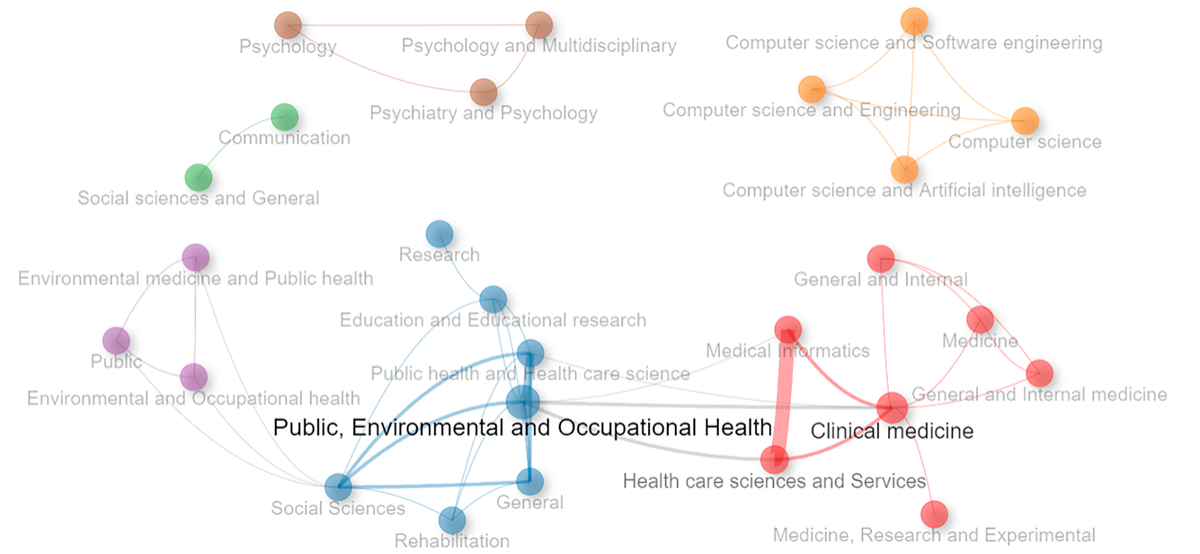
The visualization of the association network between journal categories from the master journal list of Web of Science. The connections in the network indicate how the different disciplines are thematically related based on their indexing.

Figure 2 presents a visualization of the association network between journal categories from the master journal list of Web of Science. The connections in the network indicate how the different disciplines are thematically related based on their indexing (core collection, essential science indicators, and current contents). This network is shown using a network design [39] with a Walktrap clustering algorithm and association normalization. The network includes a maximum of 50 nodes and a repulsion strength set to 0.1. As a result, 6 clusters representing different subject areas are identified: 3 of them are highly interconnected (clusters 1, 2, and 3) and 3 are isolated groups (clusters 4, 5, and 6).

Cluster 1 (blue) groups disciplines related to public, environmental, and occupational health; social sciences; rehabilitation; general research; and education and educational studies. The density of connections within this cluster suggests a strong interdisciplinary interrelation, reflecting the convergence of studies that combine public and environmental health with social and educational approaches.

Cluster 2 (red) concentrates on disciplines focused on medical informatics, health care sciences and services, and several subcategories of clinical medicine and research. The proximity and density of the interconnections suggest a strong relationship between medical research and the use of IT in health, such as medical informatics and clinical services, as well as studies in internal medicine.

Cluster 3 (purple) addresses categories related to occupational health, education, and public health. Although it is interconnected with other clusters, it maintains its distinct identity, possibly focused on issues that combine prevention and education in occupational health, as well as related public policies.

Cluster 4 (brown) is composed of categories related to psychology, psychiatry, and multidisciplinary psychology. The isolated nature of this cluster suggests that, although it is an internally interconnected field, its influence and relationship with other areas of research is limited within the analyzed network.

Cluster 5 (orange) includes categories of computer science, software engineering, artificial intelligence, and computer science and engineering. Its isolation reflects the specificity of the field and its high degree of specialization, suggesting that research topics in these areas intersect less with broader disciplines in the context of the network.

Cluster 6 (green) contains categories related to communication and social sciences in general. The isolation of this cluster could indicate that communication research, although relevant, is not strongly linked to the core areas of health and social sciences in the other larger clusters.

In the following subsections, results for each of the 4 RQs are presented.

### RQ1: How Have Pervasive Games Been Used in the Field of Sexual Health?

#### Geographic Distribution of Target Populations in Sexual Health Promotion Research

The analysis of the geographic origins of the target populations in the reviewed research articles reveals a clear concentration of studies in specific regions. The United States stands out prominently, with 50% (15/30) of the articles focusing on populations from this country, indicating a strong emphasis on American contexts and possibly reflecting the substantial resources and infrastructure available for research in sexual health promotion there. Kenya follows with 17% (5/30) of the articles, suggesting an interest in addressing sexual health issues within this East African country. India and China each contribute to the research landscape with 7% (2/30) of the articles from each country, highlighting emerging interests in sexual health promotion in South Asia and East Asia. Mexico, the Philippines, Singapore, Chile, Norway, and the United Kingdom each contributed 3% (1/30) of the articles, indicating a more limited but nonetheless notable representation from diverse geographic regions. This distribution points to a predominance of research focused on North American and African contexts, with growing attention to other global regions.

#### Audience Targeting in Sexual Health Promotion Research

The examination of the research articles revealed a varied focus on different target audiences within the realm of sexual health promotion. Adolescents, with a mean age of 14.58 (SD 0.71) years, were the most frequently addressed group, with 37% (11/30) of the articles focusing on this demographic, reflecting its significant representation and the urgent need for tailored sexual health interventions in this age group. Men who have sex with men, with a mean age of 22.29 (SD 2.64) years, were the focus of 30% (9/30) of the articles, highlighting the importance of addressing the specific health needs and risks faced by this population. Adolescents and young adults, with a mean age of 21.94 (SD 3.63) years, were featured in 20% (6/30) of the articles, indicating a recognition of the unique challenges faced by individuals in this transitional life stage. Preadolescents, with a mean age of 12.1 (SD 0.63) years, and university students, with a mean age of 20.35 (SD 1.87) years, appeared in 13% (4/30) of the articles, with 2 (7%) articles focusing on each group separately, suggesting a targeted approach to sexual health promotion in these distinct developmental and educational contexts. Although less frequently addressed, women, with a mean age of 47.5 (SD 6.64) years, and lesbian, gay, bisexual, transgender, queer, intersex, and asexual individuals were the focus of 3% (1/30) of the articles, pointing to the need for more inclusive research in these areas. This distribution underscores the diverse yet specific targeting of sexual health promotion efforts across various populations.

#### Research Dimensions for Sexual Health Promotion Through Pervasive Video Games

We observed that video games were used in 2 main areas and covered 7 topics. The first area was before the exposure, or before acquiring an STI, where video games have been developed as prevention tools. The second area was after the exposure, where video games were developed as treatment tools.

In pre-exposure category (video games as a prevention tool), we identified 6 recurring topics focusing on various aspects of STI prevention. First, we identified topics that aimed to provide general information about the impact of STIs on sexual health and the importance of protective methods, such as correct and consistent condom use and vaccination against hepatitis B (11/30, 37%). Second, we identified topics that are built as sexual health education tools, teaching about the risks of STIs; understanding one’s body, gender identity, and sexuality; building and maintaining healthy relationships; and engaging in healthy communication and decision-making about sex (10/30, 33%). Third, topics that focused specifically on promoting healthy behaviors to reduce the risk of HIV or AIDS, including decisions about whether to have sex (eg, delaying sex, abstinence, and reducing the frequency of sex), having long-term mutually monogamous relationships (eg, reducing the number of partners, avoiding concurrent partners, and increasing the time interval between sex with different partners), and consistent and correct condom use (13/30, 43%) were identified. Fourth, topics that are developed to improve understanding and uptake of pre-exposure prophylaxis medication, emphasizing the importance of preventing STIs and early diagnosis and treatment to reduce the risk of HIV transmission (3/30, 10%) were identified. Fifth, we identified topics that aimed to increase awareness about the importance of HPV vaccination and reducing the incidence of STIs, including those caused by HPV (2/30, 7%). Finally, we identified topics that focused on reducing the stigma associated with living with HIV, aiming to foster empathy in people who do not have STIs by highlighting the challenges faced by those who do (2/30, 7%).

In the postexposure category (video games as a treatment tool), we identified ART as a recurring topic, in which topics were aimed at improving adherence to ART, focusing on informing users about the benefits of ART, and including strategies for preventing HIV transmission by suppressing viral replication in people living with HIV (4/30, 13%).

#### Evolution in Time

We observed a gradual increase in scientific interest in pervasive video games focused on promoting safe sexual behaviors between the years 2015 and 2024, although with fluctuations from 1 year to another. Before 2015, only 1 study was found in this domain, with the subsequent years showing a mode of 2 papers per year. Between 2018 and 2020, significant fluctuation was observed compared to other years of research in this domain. This could suggest changes in academic priorities or the absorption and maturation of ideas introduced by these studies. The stability observed between 2020 and 2023 indicates a major interest and academic production in this domain. These results align with findings in other literature reviews, such as the study by Ndulue and Orji [22], where the trend was similar while studying multiple domains of healthy behaviors.

### RQ2: What Methods and Technologies Have Been Used to Design Pervasive Video Games for Sexual Health?

#### Overview

To our knowledge, there are no classifications or specific attributes to consider in the design, implementation, and evaluation of video games for promoting sexual health. Therefore, we subsequently propose a type of classification to organize the results in this section.

In this scenario, a general perspective on good game design is that it should specify the rules defining the world (game mechanics), user interaction with the environment (game dynamics), and the visual aspects guiding the experience (game aesthetics) [40]. Complementing this point, multiple authors affirmed that the design of the game narrative, or how events are told, directly influences the quality of the experience. In consequence, designing good storytelling guidelines and integrating them cohesively can promote a sense of personal risk or improve self-efficacy to overcome communication barriers [41-44]. They also emphasized that including game elements, such as badges, scores, or communication tables, in the design of the experience can motivate users to engage in activities they would otherwise be reluctant to perform [45]. In addition, they suggested that the incorporation of these elements in the design of the experience could improve retention and reach, promoting sustained changes over time [46]. In contrast, evaluating the effectiveness of a video game requires time and multiple studies to validate not only the robustness of the implementation but also the usability and acceptability of the experience in the population. Using the previously established definitions as a reference for the effective design, implementation, and evaluation of development, we made the classification presented in Table 1.

**Table 1:** Phases of game development.

Aspect		Description
**Design phase**		
	World rules	Describes the rules governing the game world
	User interaction	Describes how users should interact with the game world
	Aesthetics	Describes the esthetic aspects guiding the game experience
**Implementation phase**		
	Device requirements	Specifies the devices for which the experience is intended and its requirements
	Input methods	Describes the input methods used and their relationship to the game experience
	System testing	Executes the system and tests the video game with an isolated population
**Evaluation phase**		
	Population	Indicates the analyzed population and its characteristics
	Dose duration	Indicates the number of sessions and playtime per session
	Follow-up	Conducts interviews at different periods
	Measurement instruments	Describes the measurement instruments used

Considering the proposed classification listed in Table 1, the articles that were found were categorized to present a structured data format. Table 2 provides a summary of these findings. We observe that 33% (10/30) of the articles provided a detailed exposition of design, implementation, and evaluation within subcategories of the video game. On the other hand, 67% (20/30) of the studies provided partial information in these categories. For the design and its subcategories, 80% (24/30) presented all information (3/3 subcategories), 13% (4/30) partial information (2/3 subcategories), and 7% (2/30) limited information (1/3 subcategories). Then, for the implementation category and its subcategories, 57% (17/30) presented all information (3/3 subcategories), 30% (9/30) partial information (2/3 subcategories), and 10% (3/30) limited information (1/3 subcategories); also, 3% (1/30) did not present any information associated with these subcategories. Next, for the evaluation category and its subcategories, 57% (17/30) presented all information (4/4 subcategories), 37% (11/30) partial information (3/4 subcategories), and 7% (2/30) limited information (2/4 or less subcategories). Finally, it is noteworthy that all studies presented information associated with at least 2 of the 3 main categories, though the emphasis, and how deeply the topic was explored, depended on the main objective of each study. All studies incorporated some form of experimental evaluation. These findings allowed us to recognize that studies in the area of pervasive games for sexual health promotion could provide more detailed descriptions of the design and implementation processes.

**Table 2:** Classification of articles based on the presence or absence of elements of experimental design, implementation, and evaluation.

Articles	Design				Implementation				Evaluation			
	World rules	User interaction	Aesthetics	Device requirement		Inputs methods	System testing	Population		Dose duration	Follow-up	Measurement instruments
[47-51]	✓	✓	✓	✓		✓	✓	✓		✓	✓	✓
[43,52,53]	✓	✓	✓	✓		✓^a^	✓	✓		✓	✓	✓
[54]	✓	✓^a^	✓^a^	✓		✓	✓^a^	✓		✓	✓	✓
[55]	✓	✓^a^	✓	✓		✓^a^	✓^a^	✓		✓	✓	✓
[56,57]	✓	✓	✓	✓		✓	✓	✓		✓		✓
[19,58]	✓	✓	✓	✓		✓^a^		✓		✓	✓	✓
[45,59]	✓	✓	✓	✓		✓^a^	✓	✓		✓		✓
[8]	✓	✓		✓		✓	✓	✓		✓		✓
[60]	✓	✓	✓	✓		✓		✓		✓		✓
[46]	✓	✓		✓		✓		✓		✓	✓	✓
[61]	✓	✓	✓	✓				✓		✓	✓	✓
[62,63]	✓	✓	✓	✓		✓^a^		✓		✓		✓
[64]	✓	✓	✓			✓^a^	✓	✓			✓	✓
[65]	✓			✓		✓^a^	✓	✓		✓	✓	✓
[66]	✓	✓		✓^a^		✓^a^	✓	✓		✓		✓
[67]	✓			✓		✓^a^		✓		✓	✓	✓
[44]	✓	✓	✓^a^	✓^a^				✓			✓	✓
[68]	✓	✓	✓	✓		✓^a^						✓
[7]	✓	✓						✓		✓	✓	✓
[69]	✓^a^	✓^a^	✓^a^	✓				✓				✓

^a^These articles referenced these elements in a related article.

We identified 9 different methodological approaches used during the design and implementation stage. The most common approach observed was iterative development (9/30, 30%), which is a cyclical process where developers prototype an idea, conduct laboratory and user testing, and refine the game based on user feedback. The next most frequently used method was user-centered design (3/30, 10%), which is an iterative process that focuses on users and their needs at each phase of the design. This was followed by agile software development (3/30, 10%) and design thinking (2/30, 7%) methodologies, both emphasizing iterative development. Agile uses collaboration among self-organizing, cross-functional teams, and design thinking uses a problem-solving process that prioritizes user needs; it is based on empathy and hands-on iteration. Several methods were used in single instances, such as the participatory serious game development model (1/30, 3%), human-centered design (1/30, 3%), design and creation (1/30, 3%), participatory design (1/30, 3%), and embedded design (1/30, 3%). In addition, 11 video games did not specify the design method (11/30, 37%), focusing instead on presenting the research’s methodological approach. This may suggest that the design stage methodologies were considered less critical in the reporting of the development process.

#### Principal Video Game Genres

We identified and classified the video games according to their genres, listing them in descending order. The most common genre was role-playing games, which featured in 70% (21/30) of the articles. The next most popular genres were action and dating simulation, each representing 10% (3/30) of the articles. The puzzle, minigames, and education genres each appeared in 7% (2/30) of the articles. Finally, the genres casual, simulation, strategy, platformer, deck builder, and party games were each mentioned only once, accounting for 3% (1/30) of the articles in each case. One article, representing 3% (1/30) of the total articles, did not specify the game genre.

#### Physical Dimensions in Video Games: An Analysis

We identified a trend toward using 2D and 3D visuals as multimedia representations for developers and researchers. The most common dimension was 2D, appearing in 67% (20/30) of the articles. The prevalence of 2D visuals indicated a continued preference for this visual style, likely due to its straightforward graphical requirements. In contrast, 3D visuals were featured in 33% (10/30) of the articles, highlighting a significant but less frequent emphasis on more complex, immersive environments. 2.5D visual were used in 3% (1/30) of the articles, reflecting a less common but innovative approach that combines 2D and 3D graphics to create a layered visual experience. In addition, 3% (1/30) of the articles did not present the game’s physical dimensions.

#### Devices Used in Interventions

While examining the range of devices used in the interventions reported across various studies, we observed a clear predominance of smartphones. Smartphones were the most frequently used device, appearing in 80% (24/30) of the articles. This widespread use of smartphones highlights their accessibility and convenience as a platform for delivering interventions, given their ubiquity and the ease with which users can engage with content on the go. Tablets were the second most common device, featuring in 30% (9/30) of the articles, often used in conjunction with smartphones to enhance the flexibility of the intervention delivery. Computers appeared in 13% (4/30) of the articles, reflecting a preference for more traditional, larger screens in certain contexts where detailed interaction or extensive data processing might be required. In addition, web-based platforms were mentioned in 23% (7/30) of the articles, indicating a growing trend toward leveraging web environments for broader reach and interactive experiences. Finally, in 7% (2/30) of the articles, the device was not specified.

#### Analysis of Inputs and Supporting Technologies in Interventions

Analysis of inputs used across the interventions in the reviewed articles reveals a strong emphasis on multimedia and interactive elements designed to enhance user engagement and experience. A dominant input category across studies was touch screens (26/30, 87%), reflecting the versatility and the increasing reliance on touch-based interfaces for intuitive user interactions. Game controllers (game pads and joysticks) were also frequently used (21/30, 70%), highlighting their role in providing precise and interactive gameplay experiences. In addition to these common inputs, internet connection (Wi-Fi, 4G LTE, and 5G) were noted (8/30, 27%), suggesting a significant role in supporting real-time interactions and online connectivity for multiplayer or cloud-based elements. Another prevalent input was audio (7/30, 23%). This widespread use underscores the importance of immersive soundscapes in creating engaging and realistic environments for users. Artificial intelligence for nonplayable character behavior (5/30, 17%), GPS (3/30, 3%), sharing on social networks (2/30; 7%) and chatbots (2/30; 7%) were less frequent, highlighting their specialized use in certain contexts, such as location-based gaming or simulation-heavy environments. Finally, 13% (4/30) of the articles did not detail the specific inputs used.

### RQ3: What Theories are Used in the Design and Evaluation of Pervasive Games for Sexual Health?

#### Identification of Theories in the Design Stage

We were able identify 22 unique theory-based techniques used in the design stage of game development. A compilation of the observed theories and their implementation is provided in Table 3, where a breakdown of each theory, related articles, and the total number of documents using that theory are presented. It is important to note that a video game can encompass multiple theories, implying that a single document can be linked to several theories simultaneously. From the theory-based techniques observed, we noted that 90% (27/30) of the articles indicated the theory used and its relation to the research objective. We found that the social cognitive theory (SCT; 11/30, 37%) is the most recurring theory presented in the literature, followed by social learning theory (6/30, 20%), the health belief model (5/30, 17%), the theory of planned behavior (5/30, 17%), the theory of possible selves (3/30, 10%), entertainment-education literature (3/30, 10%), games for health literature (3/30, 10%) and information-motivation-behavioral skills (3/30, 10%), among others with less presence. In total, 10% (3/30) of the studies did not specify the theory upon which the video game was developed; they only stated that they were effective for behavior modification. In this case, they were categorized as “not specified” for not presenting the theory used or any support to validate its use in promoting safe sexual behaviors.

**Table 3:** Summary of theories used in the studied video games (N=30).

Theory	References	Games, n (%)
Not specified	[53,57,65]	3 (10)
Social cognitive theory	[44,45,48,49,52,55,58,64,67-69]	11 (37)
Social learning theory	[8,19,46,51,54,63]	6 (20)
Health belief model	[49,52,56,66,68]	5 (17)
Theory of planned behavior	[44,49,61,68,69]	5 (17)
Theory of possible selves	[55,58,67]	3 (10)
Entertainment-education literature	[55,58,67]	3 (10)
Games for health literature	[55,58,67]	3 (10)
Information-motivation-behavioral skills	[19,59,63]	3 (10)
Self-determination theory of motivation	[8,56]	2 (7)
Elaboration likelihood model	[60]	1 (3)
Entertainment overcoming resistance model	[62]	1 (3)
Extended parallel process model	[7]	1 (3)
Behavioral model by Fogg	[50]	1 (3)
Game-based learning and goal setting	[8]	1 (3)
Integrated model of behavior change	[50]	1 (3)
Integrative model of predictive behavior and predicted game influence	[46]	1 (3)
Motivation theory of role modeling	[8]	1 (3)
Framework of learner engagement by Prensky	[8]	1 (3)
Problem-solving theory	[48]	1 (3)
Self-efficacy message framing delay discounting	[46]	1 (3)
Yale 4Ps framework for behavior change	[8]	1 (3)
Proteus effect	[8]	1 (3)

Some examples of articles that presented the attitudinal theory used in the development of the video game and its relationship with the research objective presented are provided in Textbox 4.

**Examples of articles that include attitudinal theory in video game development and research objective.**

- Life-simulation game: based on the health belief model theory and social cognitive theory, it seeks to evaluate the effectiveness of the intervention in reducing HIV risk behaviors in an adolescent and young adult population in the United States [52].
- MyPEEPS: based on social learning theory, it aims to test the efficiency of the intervention in reducing sexual risk for HIV and promoting healthy behaviors among young men aged between 13 and 18 years [54].
- Battle Viro: based on the information-motivation-behavioral skills theory and social learning theory, it is an iPhone intervention that seeks to improve adherence to medication and antiretroviral therapy [19].
- ViralCombat: based on the social cognitive theory, theory of possible selves, entertainment-education literature, and the games for health literature, it is a second version of the Battle Viro video game that aims to improve the technical aspects of the original experience to enhance adherence to antiretroviral therapy [63].
- Tumaini: grounded in social cognitive theory and theories of narrative and applied communication, it uses a role-playing gaming experience and minigames to help young adolescents acquire the information, skills, and motivation to avoid and reduce sexual risks. The game also incorporates personal storytelling and a reward system to maintain engagement and link the gameplay to players’ real-life goals [55,58,67,68].

#### Impact of Theory-Based Techniques in the Evaluation Stage

Multiple studies have observed that teenagers and young adults are more receptive to messages related to sexuality when the information is presented in an entertaining way and comes from peers or social networks [54,57,63]. Other authors pointed out that if games are based appropriately and effectively on behavior theories, they can become valuable tools for reducing the spread of STIs [45,65] or promoting adherence to medical treatments in at-risk populations [47,59]. In addition, by building experiences that simulate risky conditions and their potential consequences, users can observe in a safe environment how STIs could influence their lives. Consequently, these experiences can help influence the determinant factors of behavior [43,68]. In contrast, authors in the field reinforced that the widespread appeal of video games among young people creates a unique opportunity to convey educational sexual messages in the users’ free time [7]. This, combined with the fact that video games are crucial for attracting and maintaining attention, makes them key components for effective behavioral change [53]. They also pointed out that this medium is ideal for promoting safe sexual behaviors outside the laboratory context, in a cost-effective and scalable manner, ensuring that the promotion is effective and not localized [19,46,56].

In more traditional contexts, where group dynamics are characterized by being focused on the family group rather than being individualistic, video games emerge as tools that provide key opportunities to extend the effects of intervention to multiple generational levels [53,67]. This interaction facilitates attitude modification, especially in contexts where gaming plays a fundamental role in the incorporation of skills related to the prevention of STIs and effective communication between parents and children [49,58]. At the same time, these experiences reduce resistance to discussing controversial topics due to age gaps, especially in contexts where support and social norms are critical for sustained behavioral change [8,50,55,67].

### RQ4: What Methods, Along With Their Benefits, Have Been Used to Evaluate Pervasive Games for Sexual Health?

#### Research Dimensions to Evaluate Sexual Health Promotion Through Pervasive Video Games

We observed that the research articles used several main methods and combined different approaches. The first and most prevalent method was the randomized controlled trial, which was used in various studies to rigorously test interventions and their effectiveness in promoting sexual health. Within this method, we identified the following frequencies: randomized controlled trial (11/30, 37%) and random clinical trial (1/30, 3%), indicating a strong emphasis on controlled experimental designs in this research area. Mixed methods research was also frequently used (11/30, 37%); this combines qualitative and quantitative approaches to provide a comprehensive understanding of the RQs. This method allows researchers to validate findings across different types of data and address complex issues from multiple perspectives. The third most common method was focus groups (9/30, 30%). This method is used to gather qualitative data, enabling researchers to explore participant experiences, perceptions, and attitudes in depth. Formative research (6/30, 20%) was used to develop and refine interventions by understanding the needs, preferences, and contexts of the target populations before full-scale implementation. Less frequently used methods included correlational studies (2/30, 7%), which examine the relationships between variables without implying causation; grounded theory (2/30, 7%), which guides the development of research based on existing theories; surveys (2/30, 7%), which collect quantitative data on participant behaviors and attitudes; and interviews (2/30, 7%), which provide in-depth qualitative insights. Several methods were used in single instances, including multimix (1/30, 3%), community-based participatory research (1/30, 3%), think aloud (1/30, 3%), descriptive research (1/30, 3%), evaluative research (1/30, 3%), participatory research (1/30, 3%), and preliminary research (1/30, 3%). Finally, 7% (2/30) of the articles did not specify the methods that were used in the evaluation stage.

This diversity of research methods highlighted the multifaceted approach to investigating sexual health promotion, using both quantitative and qualitative techniques to address the complexity of the topic across different study designs.

#### Period for Measuring the Impact of Sexual Health Interventions in Pervasive Video Games

The research on sexual health promotion through pervasive video games has used various study durations to assess the effectiveness of interventions. Short term follow-ups, from 0 to 3 months, were the most common (14/30, 47%); this allows researchers to capture immediate effects. Medium-term assessments, over 3 to 6 months (4/30, 13%), provide insights into the sustainability of intervention impacts. Long-term follow-ups, conducted over 6 to 9 months (3/30, 10%) and 10 months to ≥1 year (5/30, 17%), allow exploration of the durability of the effects. In some cases, the exact measurement periods were not specified (11/30, 37%), indicating variability in reporting practices. Pre- and postintervention outcomes (8/30, 27%) highlight the importance of understanding changes from baseline to the conclusion of the intervention.

#### Reported Benefits of Experimental Articles

In this section, only articles containing experimental data were considered; in other words, this analysis excluded articles in “protocol” format for not presenting these data. In this context, the reported benefits presented in this section only consider the 25 articles in research article format. Among the observed studies, all authors reported obtaining positive results in their experiments. We categorized the articles into 2 types: preliminary (those presenting proofs of concept and their results) and significant (those conducting statistically significant studies). Of the 25 articles, we observed that 13 (52%) were classified as preliminary, while 12 (48%) were significant. This indicates a slightly lower focus on studies with statistically significant outcomes. In addition, we found that 7 articles presented quantitative results (7/25, 28%), 10 presented qualitative data (10/25, 40%), and 8 offered mixed methods analyses (8/25, 32%). This balance suggests a robust exploration of both numerical data and in-depth insights, providing a comprehensive view of the research outcomes.

## Discussion

### Principal Findings

We reviewed 24 years of research on pervasive games aimed at promoting safe sexual behaviors. The reviewed literature suggests a positive relationship in the use of this medium for the prevention and treatment of STIs. Predominantly, research has been done in the United States (15/30, 50%) and Kenya (5/30, 17%), with growing attention across the globe. The main target population for the topic focused primarily on adolescents (11/30, 37%), men who have sex with men (9/30, 30%), and adolescents and young adults (5/30, 17%), which is in line with the priority populations for STIs from the World Health Organization [35] and Centers for Disease Control and Prevention [36].

The association network between journal categories from the master journal list of Web of Science demonstrates a strong interdisciplinary interrelation. Within the first group (highly interconnected clusters), we can appreciate a strong interdisciplinary interrelation among the 3 main clusters related to occupational health, medical informatics and education, and public health (Figure 1), which is in line with the main purpose of prevention and promotion of healthy behaviors. The second group (isolated clusters) is related mainly to psychology, computer science, and communication.

Although the focus of the first group (highly interconnected clusters) of studies is on health, in general, they are multidisciplinary in nature, reflecting how video game development for health promotion is a multidisciplinary endeavor. Furthermore, they integrate the disciplines that are isolated in the other group. However, there is room for development and research in technical terms (computer science) and psychological theories. The challenge is to generate more unified work between all disciplines to establish strong foundations in the technical, theoretical, and applied fields.

Results have shown that games for promoting safe sexual health have been used both as prevention (26/30, 87%) and treatment tools (4/30, 13%), with several approaches for prevention. In contrast, ART is a recurrent topic in this classification, showing that efforts are primarily focused on its use as a prevention tool.

Digital video games have been observed to be unique in their ability to change attitudes and behaviors in an engaging manner [61,62]. A well-designed serious game based on real-life situations serves not only to observe but also to induce behavioral changes that promote safe sexual behaviors [44].

The increasing accessibility of mobile technology provides new opportunities to implement health interventions targeted at teenagers [58,60]. This is particularly relevant in areas where discussing sex education is challenging [8,50]. The ubiquity of mobile devices allows for widespread and cost-effective prevention and skills interventions [55,67,68], and combining these technologies with persuasive approaches could improve receptivity and reduce resistance to change [7]. In this context, mobile games and web platforms have become the preferred tools for young people for sex education and behavior change [43,46,59]. The information on these platforms has the potential to influence how young people see and act [57]. Moreover, their ability to capture attention has been crucial for effective behavior change [63].

The studies presented in this review demonstrated that pervasive video games can be agents of change. However, there is a lack of work in standardizing and presenting the procedures carried out in experimental design, implementation, and evaluation. Table 2 presents a classification for each development dimension to examine what previous research has emphasized. We believe that the great variety seen in this table is natural because the field that has been developed is interdisciplinary. For example, some studies accurately determined that the use of pervasive games helps promote safe sexual behaviors, but they did not present or allow other researchers to interact with the gaming experience or understand the design and implementation, making it complex to generate new experiences using those studies as a reference, which could be interesting from a technical point of view.

These findings are consistent with other research in a similar field, where it was highlighted that the lack of standardization in mobile health apps could lead to inconsistencies in user experience and the overall effectiveness of health interventions [70]. Similarly, it was shown that the use of metadata in serious games for health not only better organizes and structures information but also increases the effectiveness of interventions [71]. Both studies highlight the importance of standardized frameworks and reinforce the need to formalize procedures in interdisciplinary fields, such as pervasive video games, emphasizing the relevance of implementing clear standards in experimental design, implementation, and evaluation stages.

In this scenario, we found that a significant number of the video games analyzed could be more easily reproduced by providing more details and characteristics about the gaming experience. Some studies could add details about the technology used, such as device specifications and input methods. This could be helped by standardizing reporting practices in a way that encourages including technological aspects rather than only focusing on content. Including technological aspects helps in the task of producing derivative versions that would enable researchers to replicate the experiment in varied environments. Consequently, adding detailed information would contribute to the ability to replicate the game experience effectively, making it easier to compare the results with findings from new studies.

Standardization is key because recognizing the vital components that affect user perception and immersion can significantly impact our comprehension of the factors influencing the experimentation process. Such insights could clarify whether issues in research findings stem from the experimental procedures or the design of the gaming experience itself.

In that sense, the need for consistency in both the design of gaming experiences and the data collection and evaluation processes becomes evident. As emphasized by Kinross [72], by implementing standardized methods, researchers can ensure that variations in user outcomes are attributable to the gaming intervention rather than inconsistencies in experimental design. This approach is essential for improving the reliability of findings in gaming research, where clear and reproducible procedures are crucial for validating the impact of game-based interventions on user perception and immersion.

We observed that 90% (27/30) of the reviewed articles used behavioral or attitudinal theories that allowed researchers to predict the outcome. This is relevant because the research and results are associated with strong theories and research from social sciences in behavioral health and not just the production of the video game.

From the theories that were found, it is notable that research has mainly concentrated on social learning theory or SCT. The most used theory was SCT, which highlights the concept of reciprocal determinism, emphasizing the interaction between individuals and their environments [73]. While many behavioral and social theories concentrate on individual, social, and environmental factors shaping behavior, SCT proposes that human behavior results from the dynamic interplay of personal, behavioral, and environmental influences [74]. Conversely, other theories focus on attitudes and their change and formation through a cognitive process.

In this context, there is a need to continue researching behavioral or attitudinal theories in other video game experiences with different designs, genres, mechanics, and so on to advance the use of mixed theories in encouraging long-term behavior. For evaluating and validating serious games for health, some guidelines are presented in the study by Kato [75], with a focus on experimental design and considerations for reporting results. Furthermore, a critical review of the literature on games for health by Verschueren et al [76] proposed a more specific framework with 5 stages (scientific, design, development, validation, and implementation) and considered all the dimensions and aspects of the development process. By covering scientific, technological, and design aspects, the review serves as an excellent guide for developing serious games based on theory for health.

We observed that the interventions reviewed mostly focused their objectives on the short and medium terms. By taking advantage of various models of behavior change, these interventions focused on reducing risky sexual behaviors, improving HIV prevention, and increasing adherence to treatments, such as pre-exposure prophylaxis and ART. For example, interventions aimed at improving sexual health knowledge, reducing stigma, and promoting health-seeking behaviors among high-risk groups have proven valuable in creating educational and engaging experiences. In contrast, little work has been done to measure the impact of long-term interventions.

These findings aligned with other research, including studies such as the study by Pouls et al [77], which emphasized the need for long-term engagement in interactive eHealth interventions to improve medication adherence over time effectively. Furthermore, as highlighted in the study by Sharifzadeh et al [78], serious games have demonstrated their capacity to influence behaviors among diverse populations, suggesting that their design can be adapted to promote sustained health outcomes. By focusing on long-term objectives, future interventions can take advantage of the engaging qualities of video games to promote continuous health-seeking behaviors while improving the effectiveness of these tools in achieving enduring behavioral changes in sexual health.

This opens space for research to demonstrate not only the capacity of video games as tools for change in the short term but also their effectiveness over time. To exemplify, the Tumaini game has undergone different stages of development and evaluation, ranging from laboratory tests to field tests with other users in various periods [55,58,67,68]. Findings from the Tumaini game consistently show positive outcomes regarding feasibility, acceptability, and effectiveness, highlighting the potential of these game-based approaches to effectively reach populations at risk. This variety of periods underlines the comprehensive approach taken to assess the effectiveness of sexual health interventions, from immediate outcomes to sustained impacts over time.

Finally, the multidisciplinary methodologies used ensure that the games are not only engaging but also grounded in sound theoretical frameworks, making them effective tools for influencing sexual health–related attitudes, intentions, and behaviors. These results reinforce the importance of innovative and accessible platforms in public health, offering a promising avenue for the continued promotion of safe sexual practices among young people. However, the studies in this review already showed promising results, with overall well-functioning technical implementation of the game elements.

### Limitations

This work might have missed some relevant articles because we made a distinction between serious games and gamified apps. Given that this study is limited to pervasive video games, several ubiquitous experiences categorized as gamified apps were excluded. This selection criterion could have omitted the analysis of certain video games in cases where the author of the original study categorized the experience as a gamified app rather than a pervasive video game and vice versa. Similarly, when categorizing articles to present a structured data format in Table 2, the definition of search terms may have omitted articles where synonyms were used or elements were described in a different way.

### Future Work and Recommendations

With the aim of driving development in the field of pervasive games, we suggest 2 directions for future research. First direction is expanding the studies to other healthy behaviors, such as mental health, tobacco cessation, and nutrition, evaluating their applicability and effectiveness in a broader spectrum. Second direction deals with working on standardizing and documenting the processes of design, implementation, and evaluation of pervasive games to improve reproducibility and facilitate access to these educational resources over time.

We recommend that researchers include a trial version or “sandbox” of their video games in their contributions. This way, other researchers can better understand how the gaming experience affects results. It would also facilitate research sustainability by providing continuous access to these resources without the need to reimplement the experience for future studies. This not only saves time but also adds long-term value to the field of study. In this context, we propose the standardization of these elements, as outlined in Table 1. This table details these aspects, emphasizing the importance of standardizing the process to advance the understanding of developments within this field.

### Conclusions

The reviewed articles suggest that pervasive video games are effective tools in promoting safe sexual behaviors, showing promising results and increasing relevance as educational tools. Their design, implementation, and evaluation are crucial factors (although not all research emphasizes these 3 dimensions), merging entertainment with education and facilitating behavior change and knowledge acquisition, especially among young people.

Pervasive video games incorporate a wide range of techniques that align effectively with theory-based techniques. Although still emerging, the results indicate that this is a growing field with considerable potential that should not be underestimated. These findings suggest that the use of pervasive video games with interactive approaches based on theories can be an effective and attractive methodology to promote education and the adoption of healthy behaviors, representing a research area with a promising future.

Finally, we encourage multidisciplinary research that integrates areas such as psychology, technology, and education to enrich both the design and effectiveness of pervasive games. We believe that the proposed approaches will not only expand knowledge in this field but also optimize the quality and effectiveness of game-based interventions.

## Appendix

Multimedia Appendix 1 PRISMA-ScR (Preferred Reporting Items for Systematic reviews and Meta-Analyses extension for Scoping Reviews) checklist.

Multimedia Appendix 2 Search strategy for each database.

Multimedia Appendix 3 Results from queries.

Multimedia Appendix 4 Full data charting.

## Abbreviations

ART: antiretroviral therapy
HPV: human papillomavirus
PICO: population, intervention, comparison, and outcomes
PRISMA-ScR: Preferred Reporting Items for Systematic Reviews and Meta-Analyses extension for Scoping Review
RQ: research question
SCT: social cognitive theory
STI: sexually transmitted infection

## Data Availability

The data are available in Multimedia Appendices 1-4.
