# Supplementary material for: Pervasive Games for Sexual Health Promotion: Scoping Literature Review
Source: JMIR Serious Games. 2025 Jan 15;13:e58912. doi: 10.2196/58912 (PMC11780293; doi:10.2196/58912)
Supplement: Multimedia Appendix 2 [file games_v13i1e58912_app2.docx]

Title: Pervasive Games for Sexual Health Promotion: Scoping Literature Review

Databases: Web of Science (WoS), Scopus, Association of Computing Machinery (ACM), and Institute of Electrical and Electronics Engineers (IEEE)

**Web Of Science**

| **Concept** | **Search Terms** | **Results** |
| --- | --- | --- |
| *#1 Videogames* | TS=(videogame OR games) | 292,572 |
| *#2 Theories and Strategies to promote or improve* | TS=(persuasive OR persuasion OR theories OR strategies OR behav* OR change OR changing OR promote OR promotion OR promoting OR attitude OR prevent OR prevention OR preventing OR improve OR improving) | 22,352,009 |
| *#3 Ubiquitous Systems* | TS = (pervasive OR ubiquitous OR mobile OR smartphone OR omnipresent OR interactive OR presence) | 4,194,001 |
| *#4 Technology and Development Steps* | TS =(design OR implement OR development OR evaluation OR technology OR technologies) | 15,605,033 |
| *#5 Health* | TS = (Health* OR wel* OR self* OR car*)) | 22,682,389 |
| *#6 Studies and Interventions of STI* | ALL=((study OR intervention) AND (sex* OR STI or infection)) | 1,940,716 |
| *#7 Combine concepts* | #1 AND #2 AND #3 AND #4 AND #5 AND #6 | 178 |
| *#8 Combine with filters* | #7 NOT Publication Years:1989; Document Types: Article; Languages: English | [150](https://www.webofscience.com/wos/woscc/summary/f758ca23-f68f-4084-b7dd-e75a7551ec0b-fff516d5/relevance/1) |

**Scopus**

| **Concept** | **Search Terms** | **Results** |
| --- | --- | --- |
| *#1 Videogames* | TITLE-ABS-KEY-AUTH ( videogame OR games) | 414,070 |
| *#2 Theories and Strategies to promote or improve* | TITLE-ABS-KEY-AUTH (persuasive OR persuasion OR theories OR strategies OR behav* OR change OR changing OR promote OR promotion OR promoting OR attitude OR prevent OR prevention OR preventing OR improve OR improving) | 4,526,351 |
| *#3 Ubiquitous Systems* | TITLE-ABS-KEY-AUTH (pervasive OR ubiquitous OR mobile OR smartphone OR omnipresent OR interactive OR presence) | 3,589,074 |
| *#4 Technology and Development Steps* | TITLE-ABS-KEY-AUTH (design OR implement OR development OR evaluation OR technology OR technologies) | 21,848,890 |
| *#5 Health* | TITLE-ABS-KEY-AUTH (Health* OR wel* OR self* OR car*) | 21,850,966 |
| *#6 Studies and Interventions of STI* | ALL((study OR intervention) AND (sex* OR STI or infection)) | 8,703,941 |
| *#7 Combine concepts* | #1 AND #2 AND #3 AND #4 AND #5 AND #6 | 741 |
| *#8 Combine with filters* | #7 AND PUBYEAR > 1999 AND PUBYEAR < 2024 AND ( LIMIT-TO ( DOCTYPE,"ar" ) ) AND ( LIMIT-TO ( LANGUAGE,"English" ) ) AND ( LIMIT-TO ( SRCTYPE,"j" ) ) | [395](https://www-scopus-com.utalca.idm.oclc.org/results/results.uri?sort=plf-f&src=s&sid=240a0ffc5a43034e71c77f8ba51e137e&sot=a&sdt=a&cluster=scosrctype%2C%22j%22%2Ct%2Bscosubtype%2C%22ar%22%2Ct%2Bscolang%2C%22English%22%2Ct&sl=577&s=TITLE-ABS-KEY-AUTH+%28+%28+videogame+OR+games+%29+AND+%28+persuasive+OR+persuasion+OR+theories+OR+strategies+OR+behav*+OR+change+OR+changing+OR+promote+OR+promotion+OR+promoting+OR+attitude+OR+prevent+OR+prevention+OR+preventing+OR+improve+OR+improving+%29+AND+%28+pervasive+OR+ubiquitous+OR+mobile+OR+smartphone+OR+omnipresent+OR+interactive+OR+presence+%29+AND+%28+design+OR+implement+OR+development+OR+technology+OR+technologies+%29+AND+%28+health*+OR+well*+OR+self*+OR+care*+%29+%29+AND+ALL+%28+%28+study+OR+intervention+%29+AND+%28+sex*+OR+sti+OR+infection+%29+%29+AND+PUBYEAR+%26gt%3B+1999+AND+PUBYEAR+%26lt%3B+2024&origin=searchadvanced&editSaveSearch=&txGid=41df3924387388e5b2546d12b82cffaf&sessionSearchId=240a0ffc5a43034e71c77f8ba51e137e&limit=10) |

**IEEE**

| **Concept** | **Search Terms** | **Results** |
| --- | --- | --- |
| *#1 Videogames* | (videogame OR games) | 83,148 |
| *#2 Theories and Strategies to promote or improve* | (persuasive OR persuasion OR theories OR strategies OR behav* OR change OR changing OR promote OR promotion OR promoting OR attitude OR prevent OR prevention OR preventing OR improve OR improving) | 2,500,500 |
| *#3 Ubiquitous Systems* | (pervasive OR ubiquitous OR mobile OR smartphone OR omnipresent OR interactive OR presence) | 713,185 |
| *#4 Technology and Development Steps* | (design OR implement OR development OR evaluation OR technology OR technologies) | 4,053,891 |
| *#5 Health* | (Health* OR wel* OR self* OR car*) | 21,850,966 |
| *#6 Studies and Interventions of STI* | (study OR intervention) AND (sex* OR STI or infection) | 2,524 |
| *#7 Combine concepts* | #1 AND #2 AND #3 AND #4 AND #5 AND #6 | 741 |
| *#8 Combine with filters* | #7 AND Journals | [2](https://ieeexplore-ieee-org.utalca.idm.oclc.org/search/searchresult.jsp?action=search&matchBoolean=true&queryText=((videogame%20OR%20games)%20AND%20(persuasive%20OR%20persuasion%20OR%20theories%20OR%20strategies%20OR%20behav*%20OR%20change%20OR%20changing%20OR%20promote%20OR%20promotion%20OR%20promoting%20OR%20attitude%20OR%20prevent%20OR%20prevention%20OR%20preventing%20OR%20improve%20OR%20improving)%20AND%20(pervasive%20OR%20ubiquitous%20OR%20mobile%20OR%20smartphone%20OR%20omnipresent%20OR%20interactive%20OR%20presence)%20AND%20(design%20OR%20implement%20OR%20development%20OR%20technology%20OR%20technologies)%20AND%20(Health*%20OR%20well*%20OR%20self*%20OR%20care*)%20AND%20((study%20OR%20intervention)%20AND%20(sex*%20OR%20STI)))&highlight=true&returnFacets=ALL&returnType=SEARCH&matchPubs=true&rowsPerPage=25&refinements=ContentType:Journals) |

**ACM Full – Text collection**

| **Concept** | **Search Terms** | **Results** |
| --- | --- | --- |
| *#1 Videogames* | (videogame OR games) | 23,781 |
| *#2 Theories and Strategies to promote or improve* | (persuasive OR persuasion OR theories OR strategies OR behav* OR change OR changing OR promote OR promotion OR promoting OR attitude OR prevent OR prevention OR preventing OR improve OR improving) | 333,403 |
| *#3 Ubiquitous Systems* | (pervasive OR ubiquitous OR mobile OR smartphone OR omnipresent OR interactive OR presence) | 293,448 |
| *#4 Technology and Development Steps* | (design OR implement OR development OR evaluation OR technology OR technologies) | 448,500 |
| *#5 Health* | (Health* OR wel* OR self* OR car*) | 168,144 |
| *#6 Studies and Interventions of STI* | (study OR intervention) AND (sex* OR STI or infection) | 18,215 |
| *#7 Combine concepts* | #1 AND #2 AND #3 AND #4 AND #5 AND #6 | 105 |
| *#8 Combine with filters* | #7 AND [E-Publication Date: (01/01/2000 TO 08/31/2024)] AND Content Type: Research Article | [76](https://dl.acm.org/action/doSearch?fillQuickSearch=false&target=advanced&expand=dl&field1=Abstract&text1=%28videogame+OR+games%29+AND+%28persuasive+OR+persuasion+OR+theories+OR+Strategies+OR+behav*+OR+change+OR+changing+OR+promote+OR+promotion+OR+promoting+OR+Attitude+OR+prevent+OR+prevention+OR+preventing+OR+improve+OR+improving%29+AND+%28pervasive+OR+ubiquitous+OR+mobile+OR+Smartphone+OR+omnipresent+OR+everywhere+OR+interactive+OR+presence%29+AND+%28design+OR+implement+OR+development+OR+evaluation+OR+technology+OR+technologies%29+AND+%28health*+OR+wel*+OR+self*+OR+car*%29&field2=AllField&text2=%28study+OR+intervention%29+AND+%28sex*+OR+STI+or+infection%29&searchArea%5B0%5D=SeriesKey&operator%5B0%5D=And&text%5B0%5D=&EpubDate=&AfterMonth=1&AfterYear=2000&BeforeMonth=8&BeforeYear=2024&startPage=&ContentItemType=research-article) |
